# Association of postnatal age with neonatal hospital-onset bacteremia in a multicenter, retrospective cohort

**DOI:** 10.1017/ash.2023.365

**Published:** 2023-09-29

**Authors:** Erica Prochaska, Shaoming Xiao, Elizabeth Colantuoni, Sagori Mukhopadhyay, Dustin Flannery, Ibukun Kalu, Danielle Zerr, Amanda Adler, Aaron Milstone

## Abstract

**Background:** Prevention of hospital-onset bacteremia (HOB) in all settings is a healthcare priority. The CDC is developing a neonatal-specific HOB quality metric, but the epidemiology of neonatal HOB is poorly understood. Our objective was to validate a prior single-center finding that HOB risk varies by birthweight and postnatal age in a multicenter cohort. **Methods:** We performed a multicenter, retrospective cohort study of neonates admitted to 4 neonatal intensive care units (NICUs) for ≥4 days between July 1, 2016, and July 1, 2021. HOB was defined as a positive blood culture for bacteria or fungi on day ≥4 of admission. The first HOB event in the hospitalization was counted per neonate. Repeat HOB events during a neonate’s admission were excluded. Poisson regression models with robust variance estimates were used to estimate the incidence rate (IR) of HOB, expressed as HOB events per 1,000 patient days and IR ratios (IRRs), within strata defined by CDC birthweight categories and 4-week postnatal age intervals, adjusting for central venous catheter (CVC) presence at time of HOB and study site. **Results:** The analysis included 9,267 neonates, contributing 191,295 patient days and 470 HOB events, with an unadjusted IR of 2.46 per 1,000 patient days (Table 1). Of 477 infants born ≤750 g, 153 (30.1%) had a HOB with an IR of 13.3 (95% CI, 10.5–16.0) events per 1,000 patient days in the first 4 weeks after birth (Fig. 1). After adjusting for CVC presence and study site, infants ≤750 g had a higher HOB rate in the first 4 weeks of life (IRR, 7.45; 95% CI, 3.81–14.56) compared to infants ≥2,500 g. After 8 weeks of life, there was no difference in HOB rate in the 2 groups (IRR, 0.8, 95% CI, 0.3–2.7). **Conclusions:** Neonates born ≤750 g were at highest risk for HOB within the first 4 weeks after birth; however, risk for HOB was not consistent over time. Postnatal age should be considered in a neonatal HOB quality metric.

**Figure:**
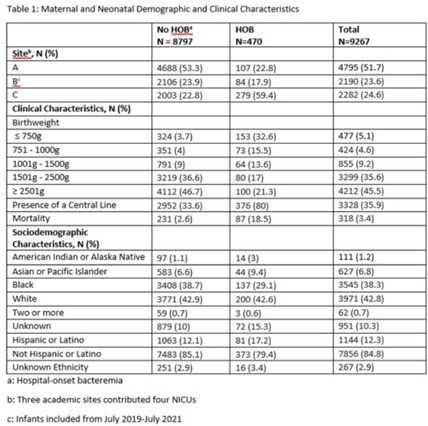

**Figure:**
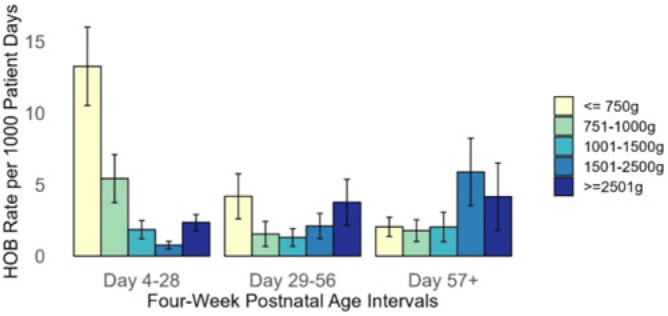

**Disclosures:** None

